# Screening for *in vitro* systematic reviews: a comparison of screening methods and training of a machine learning classifier

**DOI:** 10.1042/CS20220594

**Published:** 2023-01-27

**Authors:** Emma Wilson, Florenz Cruz, Duncan Maclean, Joly Ghanawi, Sarah K. McCann, Paul M. Brennan, Jing Liao, Emily S. Sena, Malcolm Macleod

**Affiliations:** 1 Centre for Clinical Brain Sciences, The University of Edinburgh, Edinburgh, U.K.; 2 Berlin Institute of Health at Charité-Universitätsmedizin Berlin, QUEST Center, Berlin, Germany; 3 University of Edinburgh Medical School, University of Edinburgh, Edinburgh, U.K.; 4 Independent Researcher, U.K.

**Keywords:** automation, in vitro models, machine learning, Meta-research, systematic review

## Abstract

Objective: Existing strategies to identify relevant studies for systematic review may not perform equally well across research domains. We compare four approaches based on either human or automated screening of either title and abstract or full text, and report the training of a machine learning algorithm to identify *in vitro* studies from bibliographic records. Methods: We used a systematic review of oxygen–glucose deprivation (OGD) in PC-12 cells to compare approaches. For human screening, two reviewers independently screened studies based on title and abstract or full text, with disagreements reconciled by a third. For automated screening, we applied text mining to either title and abstract or full text. We trained a machine learning algorithm with decisions from 2000 randomly selected PubMed Central records enriched with a dataset of known *in vitro* studies. Results: Full-text approaches performed best, with human (sensitivity: 0.990, specificity: 1.000 and precision: 0.994) outperforming text mining (sensitivity: 0.972, specificity: 0.980 and precision: 0.764). For title and abstract, text mining (sensitivity: 0.890, specificity: 0.995 and precision: 0.922) outperformed human screening (sensitivity: 0.862, specificity: 0.998 and precision: 0.975). At our target sensitivity of 95% the algorithm performed with specificity of 0.850 and precision of 0.700. Conclusion: In this *in vitro* systematic review, human screening based on title and abstract erroneously excluded 14% of relevant studies, perhaps because title and abstract provide an incomplete description of methods used. Our algorithm might be used as a first selection phase in *in vitro* systematic reviews to limit the extent of full text screening required.

## Introduction

Experiments conducted *in vitro* play an invaluable role in the research pipeline. *In vitro* models, including 3D organoids, have recently attracted attention as methods which might reduce and eventually replace the use of animals in research [1]. However, challenges in translating findings from *in vitro* research to the clinic may hinder efforts to replace animal research. Poor reporting of measures to reduce the risk of bias in *in vitro* studies may contribute to this translational challenge [2], and research which systematically identifies such issues [3] may lead to improvements in the design, conduct and reporting of *in vitro* research, and, thereby, their adoption as alternatives to animal research.

Systematic review is a research method used to summarise and critically appraise all available published evidence related to a pre-defined research question [4]. The use of systematic review to evaluate evidence from clinical trials has led to significant improvements in clinical trial design, conduct and reporting [5]. The application of systematic review methodologies to *in vivo* animal studies has, similarly, identified opportunities for improvement [6,7]. More recently, reviews of *in vitro* data have suggested similar problems may be prevalent there [2,3,8].

Tools and guidance developed by Cochrane have contributed substantially to improving the methodological quality of clinical systematic reviews [9–12]. Similar guidance has been articulated for systematic reviews of animal studies including a protocol template [13], the CAMARADES reporting quality checklist [14], the SYRCLE risk of bias checklist [15], and the development of a Preferred Reporting Items for Systematic reviews and Meta-Analyses (PRISMA) extension for such reviews is ongoing [16]. These adapted guidelines reflect important differences between clinical and animal studies, including study size (many human patients per study versus few laboratory animals per study) and heterogeneity between studies (lower in human than in animal studies).

It is possible that the methods used to plan and conduct *in vitro* systematic reviews must be further adapted. One key feature is the process of searching and screening for relevant publications. In a typical systematic review of animal data, search results from multiple databases are combined, duplicate citations are removed, and titles and abstracts are screened for relevance. General guidance is that the screeners should, if anything, be over-inclusive at this stage (i.e. perform with high sensitivity, perhaps at a cost in specificity [17,18]). This stage is followed by the full-text screening to determine eligibility.

In a pilot systematic review of *in vitro* data conducted in 2019 (unpublished), we found an unexpectedly low yield of included studies and hypothesised that title and abstract ([TiAb]) screening may not be sufficiently sensitive. Where animals and *in vitro* experiments were reported in the same publication, we were concerned that a full summary of *in vitro* methods and results may not always be included in the abstract. This would lead to studies being incorrectly excluded at the [TiAb] screening phase. Further, as systematic searches are often conducted on [TiAb] text – especially where relevant field tags such as MeSH terms may not be available – relevant *in vitro* studies may not even be identified in literature searches specifically designed to identify *in vitro*-related terms. These concerns are consistent with a recent finding that, in studies where multiple outcomes were investigated, negative findings were less likely to be included in the abstract text and, therefore, less likely to be included in systematic reviews [19]. In our view, for the purposes of most systematic reviews, screening approaches should perform with a sensitivity of at least 95%, that is, they should wrongly exclude fewer than 1 in 20 relevant studies.

One approach to this problem would be to conduct broader systematic searches to capture any article that might contain an *in vitro* experiment and to screen studies for relevance on the basis of the full-text PDF article. However, this would be significantly burdensome, in a context where a major limitation of current methodologies is the time and effort required to complete a systematic review. This is especially true in preclinical systematic reviews, which tend to screen and include a higher number of publications compared with clinical reviews.

Recently, automation tools have been developed to accelerate parts of the systematic review process including screening [20–22], PICO extraction [23,24] and risk of bias assessment [25–27]. These tools allow researchers to conduct reviews more quickly and without requiring as much human effort; we wondered if automation tools might address the issue of incomplete [TiAb] descriptions.

### Aims

Here, we compare the performance of four different screening methods – (i) human screening based on [TiAb] only, (ii) human screening based on full text, (iii) automated screening based on [TiAb] only and (iv) automated screening based on full text – in an exemplar systematic review of ischaemic injury induced by oxygen–glucose deprivation in PC-12 cells. Then, we train a machine learning algorithm, developed specifically for systematic review screening, to identify studies which report the results of *in vitro* experiments.

## Methods

### Method 1: Comparison of screening methods in an example systematic review

The study protocol for the comparison of screening methods is available at https://osf.io/cq48b/. Methods of analyses were not described in the protocol, and deviations from the protocol are described in Appendix 1.

#### Search strategy

We conducted a systematic search of PubMed (accessed via NCBI) and Embase (accessed via Ovid) on 16 March 2020. Full search terms are given in Appendix 2(i) and included a series of terms to identify the experimental approach (e.g. ‘oxygen–glucose deprivation’), the condition modelled (e.g. ‘brain ischaemia’) and the experimental materials (e.g. ‘PC-12’). An error in implementing the search terms led to our combining the first two of these phrases with ‘OR’ rather than ‘AND’ (the errors are underlined in Appendix 2(i)) resulting in the retrieval of many more studies than had the search been implemented as intended. We did not notice this error until all studies had been screened, and we provide a primary analysis of the search as implemented, with a secondary analysis of the search as was intended.

We imported each search result into EndNote X8, created a single XML file of all search results, and removed duplicate citations using the Automated Systematic Search Deduplicator (ASySD) tool [28]. This performs automatic deduplication with limited human input and was designed specifically for use in preclinical (but not necessarily *in vitro*) systematic review projects. We imported our deduplicated search results to EndNote X8 and retrieved full text PDFs using EndNote’s in-built ‘find full text’ feature, then converted PDFs to plain text files using the PDF to text function from XpdfReader (https://www.xpdfreader.com/).

#### Eligibility criteria for analysis

We included records which had both an English-language abstract and an English-language full text. We excluded conference abstracts, records with no abstract, records with no English-language full text, records where a full text was not retrieved by EndNote X8, and records which did not have a machine-readable full text.

#### Systematic review Inclusion and exclusion criteria

The screening task was to identify controlled experiments exposing PC-12 cells to oxygen–glucose deprivation (OGD) *in vitro* and reporting effects on cell death or survival (MTT assay, LDH assay, or cell counting), whether investigating the effects of OGD or the impact of interventions (e.g. pharmacological and genetic) intended to modulate the effects of OGD. There was no limitation by publication date.

#### Human screening

For human screening, we used the Systematic Review Facility (SyRF) web application [29] to screen studies against our inclusion criteria. A pool of six reviewers were allocated records in random order, and each record was screened by at least two reviewers. Where there was disagreement, the record was automatically presented to a third reviewer for arbitration. All decisions were taken blinded to the decision(s) of other reviewers, and whether the task was initial screening (i.e. ‘reviewer 1’ or ‘2’) or reconciliation of conflicting opinions (‘reviewer 3’). Reviewers first screened each study based on [TiAb], and then, in the same session, were asked to screen the study again based on the full-text PDF. Therefore, each publication was screened twice, first on the basis of [TiAb] and then on the basis of the full text.

#### Automated screening using regular expressions

For automated screening, we used the R programming language and Regular Expressions (RegEx). A regular expression is a sequence of characters which can be used to search for and match certain patterns within text [30]. We developed a RegEx to identify relevant publications by matching terms such as ‘oxygen–glucose deprivation’, ‘OGD’, ‘oxygen and glucose deprivation’ or ‘deprived of oxygen and glucose’. The full RegEx is given in Appendix 3. We then used the AutoAnnotation R package [31] to count the number of occurrences of regular expressions matches in the [TiAb] or full text. One match meant that some form of the term oxygen–glucose deprivation was mentioned only once within the text, two matches meant that some form of the term was mentioned twice, etc.

#### The gold standard dataset

To create a dataset with the highest proportion of true decisions, we reasoned that reconciled human full-text screening decisions were likely to be most complete. Where there was disagreement between the human full-text decision and another decision, then that study was evaluated by a senior experienced reviewer, and where they were not in agreement with the reconciled human full-text screening decision, their re-evaluated decision was used as the gold standard.

#### Evaluation of screening performance

We assessed the performance of each approach by calculating the sensitivity, specificity and precision, characterising the ‘purity in retrieval performance’ [32], (number of true positive decisions divided by the number of positive decisions) using the Caret R package [33].

#### Assessing best performance

Perfect performance is achieved when sensitivity and specificity are both 100%. A total of 100% sensitivity is achieved when all relevant publications are included during screening, and 100% specificity is achieved when all non-relevant publications are excluded during screening. We calculated the Euclidian distance (*d*) between the performance achieved and this optimum performance as

$$d=\sqrt{{\left(1-Sensitivity\right)}^{2}+{\left(1-Specificity\right)}^{2}}$$

and the screening method with the smallest value of *d* was considered optimal. For the automation approaches, we used the same approach to calculate the point on the receiver operating characteristic (ROC) curve closest to peak performance. As a second measure of performance, we used the area under the ROC curve.

### Method 2: Developing a trained machine learning classifier for *in vitro* systematic review screening

The study protocol for the development of a machine learning classifier is available at https://osf.io/bjsp2/. Deviations from the protocol methods are described in Appendix 1.

#### Definition of *in vitro* research

For the purposes of developing this screening tool, we define *in vitro* research as involving the manipulation of biomolecules (including enzymes, genes and genomes), cells, tissues, or organs in a controlled, artificial environment such as a Petri dish, well or test tube.

Our definition includes samples which may also be described as *ex vivo* (tissues originating from experimental animals) if the experimental intervention under investigation was applied to the specimen after derivation rather than being applied *in vivo* pre mortem or before tissue collection.

#### Generation of a screened dataset

Using the PMC API, we downloaded 2,000 randomly sampled records from PubMed Central (PMC) on the 19 December 2019 [34]. We used no search terms, filters or restrictions to generate this sample.

We uploaded all 2000 PMC records to the SyRF web application for full text screening based on our definition of *in vitro* research, given above. Each study was screened by two independent reviewers and disagreements were reconciled by a third independent reviewer.

We then supplemented our 2000 screened records with 453 known *in vitro* studies previously screened as part of the Nature Publication Quality Improvement Project (NPQIP) study [2]. The merged dataset included a unique identifier for each study, the [TiAb] text and a binary flag indicate the include or exclude screening decision.

#### Training the machine learning algorithm

We used the binary screening decisions (‘include’ or ‘exclude’) from our merged dataset to train a machine learning algorithm hosted by our collaborators at The Evidence for Policy and Practice Information and Co-ordinating Centre (EPPI-Centre), University College London. The algorithm uses a tri-gram ‘bag-of-words’ model for feature selection and implements a linear support vector machine (SVM) with stochastic gradient descent (SGD), as described in Approach 1 used by Bannach-Brown et al. [21]. The algorithm associates the training set screening decisions with features it identifies in the relevant [TiAb] text, and uses these features to predict the inclusion or exclusion status for new unseen studies.

The dataset was randomly split into training set (80%) and validation set (20%) to ensure the algorithm performed optimally.

#### Error correction and retraining classifier

After algorithm training, we performed a round of error correction as described by Bannach-Brown et al. [21]. We identified the 100 studies with the largest discrepancy between human screening and algorithm score, and had humans rescreen these studies to identify if there had been a human error during screening. We then retrained the machine learning algorithm using the set of 2453 screened records thus corrected.

## Results

### Performance of different screening methods for case study: *in vitro* OGD systematic review

#### Search results

**Figure 1:**
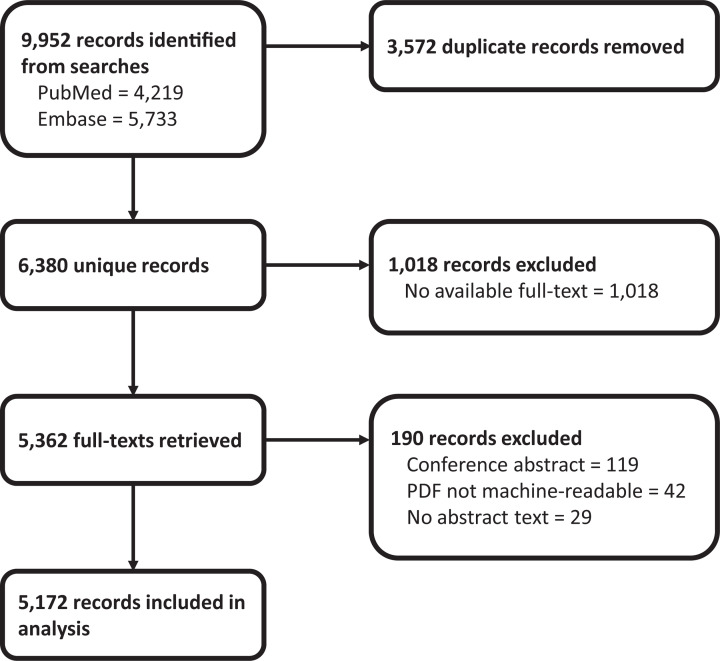
Flowchart of records retrieved from systematic searches, full texts retrieved, and records included in screening comparison analysis

Figure 1 shows the PRISMA flow diagram. Our systematic search as implemented retrieved a total of 9952 records (4219 from NCBI PubMed and 5733 from Ovid Embase). Following deduplication, we identified 6380 unique records.

We were able to retrieve full-text PDFs for 5362 (84%) of the unique records identified from our search. From this, we included a total of 5172 records in our analysis. We excluded 119 records which where conference abstracts, 42 records where the PDF was not machine-readable, and 29 records which had no abstract.

#### Performance of different screening methods

**Figure 2:**
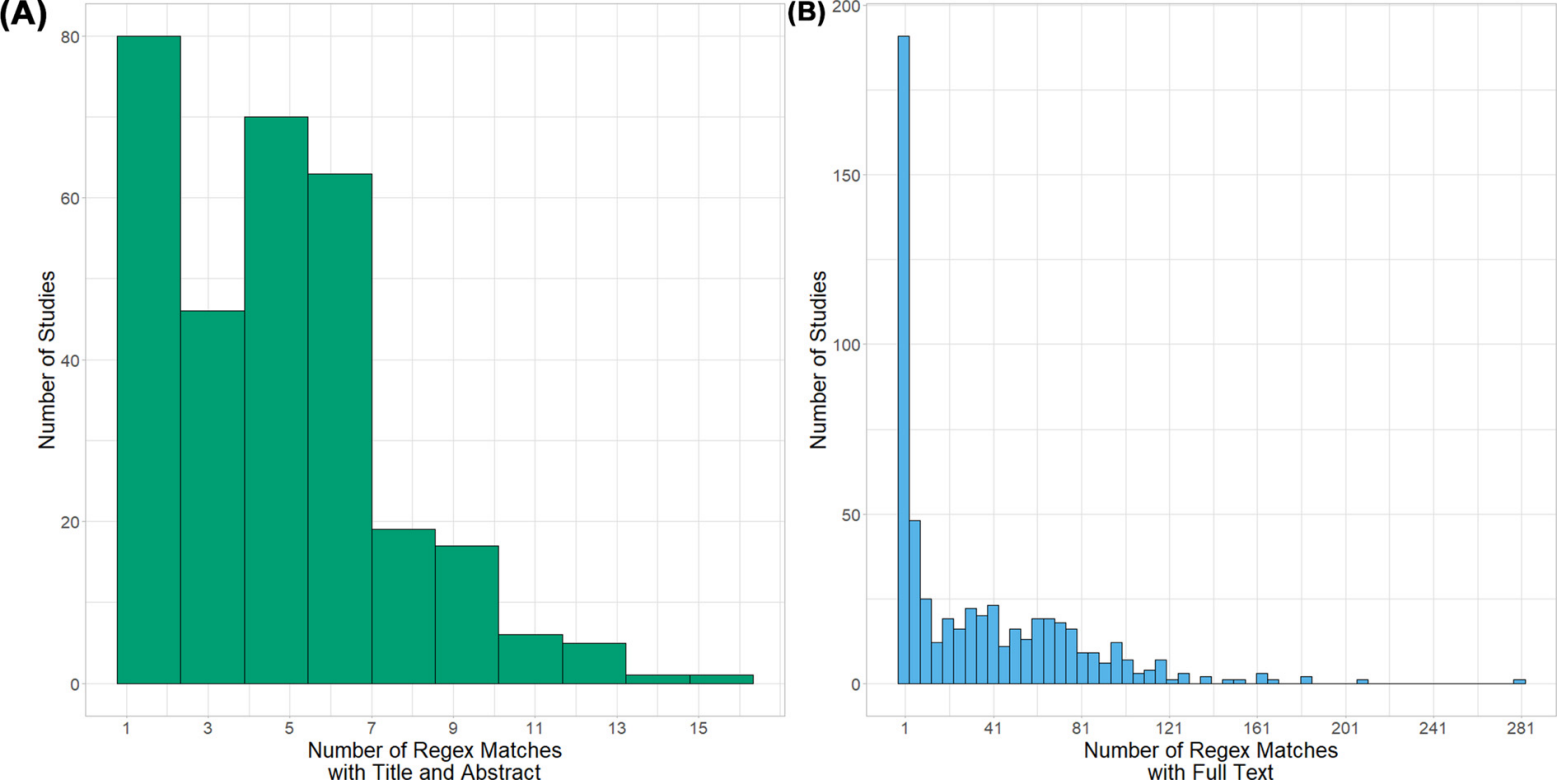
Histograms showing the number of studies against the number of regex matches with (A) title and abstract and (B) full text. Histograms showing the number of studies against the number of regex matches with (**A**) title and abstract and (**B**) full text.

**Figure 3:**
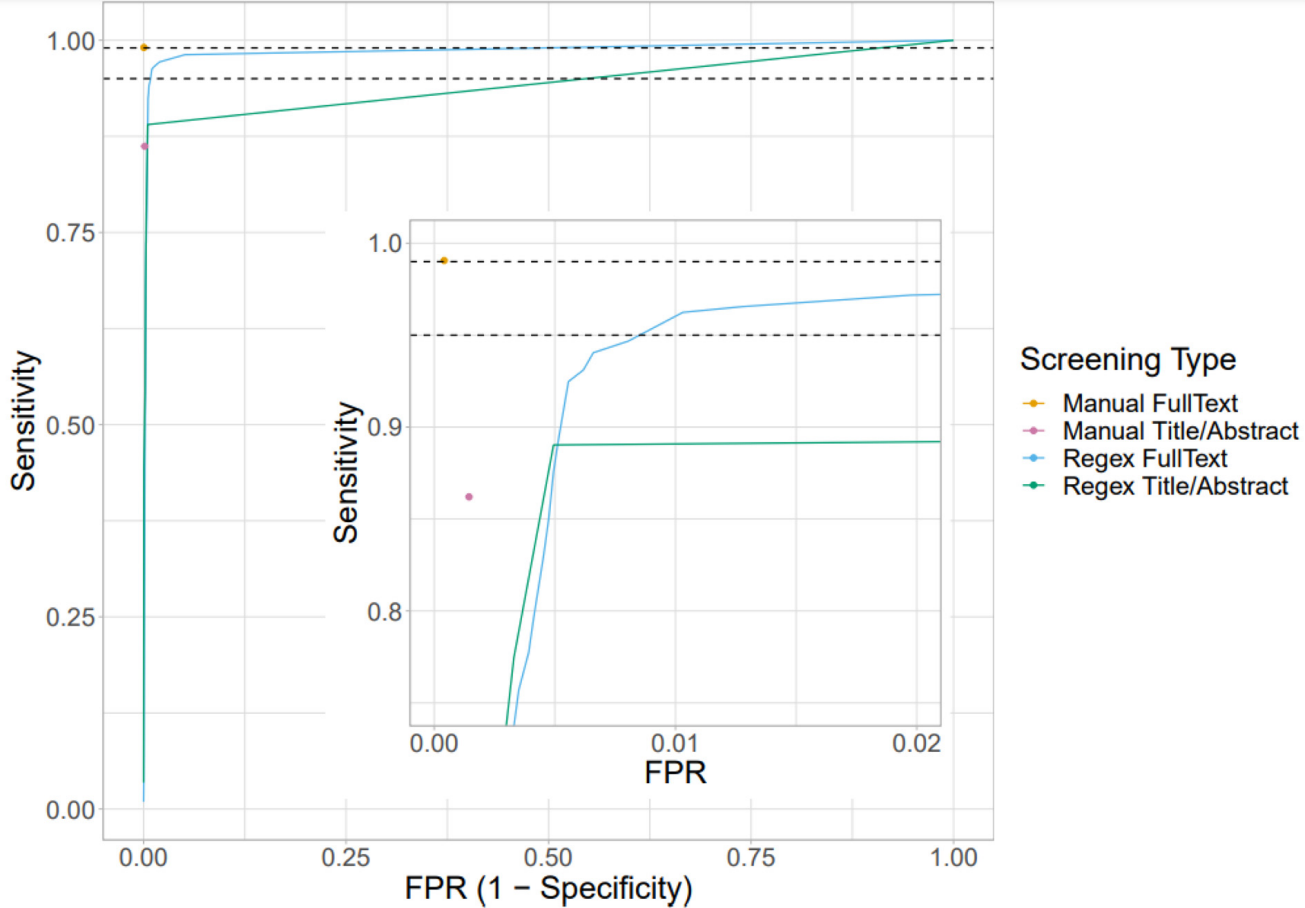
Receiver Operating Characteristic (ROC) curve showing the performance of all screening types at all thresholds Horizontal dashed lines show 99% (0.99) and 95% (0.95) sensitivity. FPR = false positive rate. Inset shows the top left of the graph in more detail.

Human reviewers identified 282 of 5172 records for inclusion based on [TiAb], and 318 of 5172 when screening against full text. The number of RegEx matches was between 0 and 15 for [TiAb], and between 0 and 281 for full text (Figure 2). We then calculated the sensitivity and specificity at each RegEx threshold (i.e. including studies based on *N* RegEx matches, with *n* = 1–281) and set thresholds for inclusion of 1 match for [TiAb] screening and two matches for full-text screening (Figure 3). Finally, we re-examined those records where there was a discrepancy between human full-text screening and one of the other screening approaches. This focussed review identified three records which had been omitted by human full text screening but identified by the full-text RegEx, and two records included in error by human full-text screening. This gave 319 included studies (6.2% of 5172).

Compared with this gold standard, human [TiAb] screening correctly identified 275 of 319 studies, and wrongly included an additional seven studies (*d* = 0.138). Human full-text screening correctly identified 316 of 319 studies, wrongly including two studies (*d* = 0.009). RegEx of [TiAb] correctly identified 284 of 319 studies and wrongly included 24 studies (*d* = 0.110), and RegEx of full text (with an optimal threshold of two matches) correctly identified 310 of 319 studies, wrongly including 96 (*d* = 0.034) (Table 1). Area under the curve (AUC) was 0.944 for RegEx applied to [TiAb] and 0.986 for RegEx of full text.

**Table 1:** Performance of different screening methods

Screening method	Number of true positives	Number of true negatives	Number of false positives	Number of false negatives	Sensitivity	Specificity	Precision	Euclidian distance *(d)*	AUC
Human Title/Abstract	275	4846	7	44	0.862	0.998	0.975	0.138	0.930
Human Full text	316	4851	2	3	0.990	1.000	0.994	0.009	0.995
RegEx Title/Abstract	284	4829	24	35	0.890	0.995	0.922	0.110	0.944
RegEx Full text	310	4757	96	9	0.972	0.980	0.764	0.034	0.986

A total of 5172 records were screened using each method. For sensitivity, specificity and precision, the optimal performance value is 1. For RegEx title/abstract, the optimal threshold shown is 1 match. For RegEx full text, the optimal threshold shown is two matches. A lower Euclidian distance (*d*) indicates better performance.

A total of 1060 citations were excluded from the full-text analysis because we were unable to retrieve (1018) or to process (42) the full text. Within this additional corpus, human [TiAb] and RegEx [TiAb] screening, respectively, identified 57 and 66 additional studies which appeared relevant. Without access to the full text, we cannot determine how many of these might be false positives, and given the sensitivity of these approaches in the main cohort of studies, it is likely that further relevant studies will have been excluded.

#### Analysis of the ‘intended’ search strategy

The error in implementing our search strategy had a profoundly beneficial effect on our ability to detect relevant articles. On 5 May 2022, we searched NCBI PubMed and Ovid Embase using our intended search strategy (Appendix 2(ii)), limited by date of record creation to 16 March 2020 (to align with the initial search), and retrieved 910 unique records (438 from NCBI PubMed and 700 from Ovid Embase, compared with 4219 and 5733, respectively, in the ‘incorrectly’ implemented search). Remarkably, only 133 (or 42%) out of the 319 studies we identified using our ‘incorrect’ search were identified by our planned search strategy. If we had used this approach, and if subsequent human [TiAb] had been conducted, the performance of human [TiAb] screening would have been overinflated, giving an apparent sensitivity of 0.925 and specificity of 0.999.

### Training a machine learning classifier for *in vitro* systematic review screening

#### Dataset of screened studies

Of the 2000 articles randomly selected from PMC, after full text screening we judged 296 to describe *in vitro* research. Combining these with 453 *in vitro* studies from NPQIP, gave a complete dataset with 749 included studies and 1704 excluded studies (total *n*=2453). We randomly divided these into training (*n*=1962) and validation (*n*=491) sets.

#### Machine learning performance

**Figure 4:**
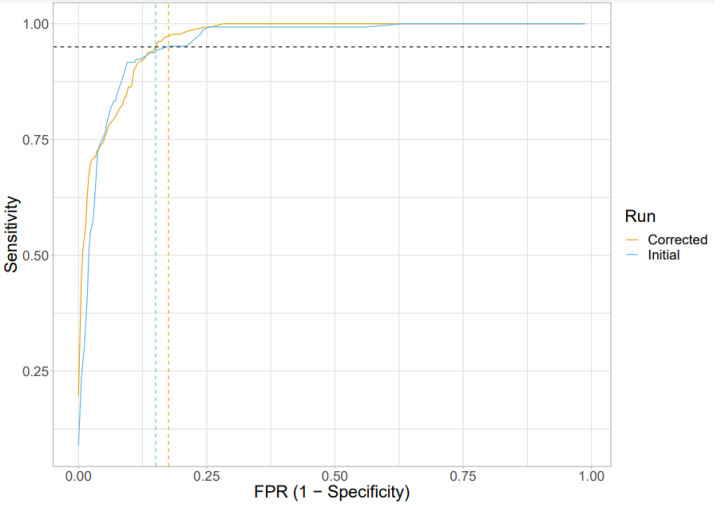
ROC curve showing both the initial and corrected performance of the machine learning algorithm at all thresholds The vertical dashed lines show the optimal threshold (0.25 for the initial performance and 0.29 for the corrected performance); FPR, false positive rate.

We trained the machine learning algorithm on title and abstract [TiAb] in the training set, and then applied the algorithm to the validation set, attributing each citation with a decimal score between 0 and 1, where higher scores reflect a stronger machine prediction of inclusion. We then established a threshold such that 95% of relevant studies in the validation set would be retrieved (i.e. sensitivity = 0.950 or higher). A machine score threshold for inclusion of 0.25 (Figure 4) gave specificity of 0.824 at sensitivity of 0.951 and precision of 0.692 (Table 2). We then checked human decisions for the 100 citations with greatest mismatch between human decisions and machine predictions. A total of 35 citations had the human decision reversed, with 31 citations included by human decision now excluded, and four citations excluded by human decision now included. Retraining on this revised corpus gave specificity of 0.850 (increase in 0.026) at sensitivity of 0.954, and precision of 0.700 (increase in 0.008) (Table 2), with a machine score threshold for inclusion of 0.29 (Figure 4).

**Table 2:** Performance of the trained machine learning algorithm before and after error correction

	Sensitivity	Specificity	Precision
**Initial**	0.951	0.824	0.692
**Corrected**	0.954	0.850	0.700

## Discussion

### Screening in *in vitro* systematic reviews

In typical biomedical systematic reviews, a systematic search of [TiAb] text retrieves potentially relevant articles, which are then screened by two independent reviewers, and any disagreements reconciled by a third reviewer. The broader the terms of the systematic search the higher will be the sensitivity, but because of the inevitably high total number of citations returned, this will come at the cost of an increased burden of human screening. Here, we show that in a systematic review of the effects of OGD in PC-12 cells, human screening of [TiAb] was the least sensitive (0.862) of four approaches tested and would have wrongly excluded around one in every seven relevant manuscripts. Human full text screening performs with a sensitivity of 0.990, wrongly excluding only 1 in 1000 manuscripts. However, because of the time involved, this is not a feasible approach for most systematic reviews.

While we did not formally compare the time taken by human reviewers and the RegEx algorithms, there is a substantial reduction in time taken, even accounting for the requirement to develop the regular expressions and convert PDF to text. Dual human screening of 5000 [TiAb], even at 30 s per record, would take over 80 h, and full text screening around 800 h, compared with less than one day for the RegEx approach.

The RegEx approach achieved higher sensitivity than human screening when applied to [TiAb] text. For full text, sensitivity was slightly lower (0.972) than human screening (0.990). For both RegEx approaches, specificity was lower than human screening ([TiAb]: 0.995 versus 0.998: full text 0.980 versus 1.000). For contrast with human [TiAb] screening, RegEx full-text screening identifies an extra 35 studies (13%) which should be included, at a cost of increasing the number included in error from 7 to 96. This could, therefore, serve as a useful first step before human full text screening, which could take place at the data extraction stage. However, the usefulness of RegEx full-text screening will be heavily dependent on the quality of that RegEx, and we strongly advise researchers carefully to consider synonyms, alternate spellings and different combinations of target words or phrases.

The benefits of this approach were highlighted, inadvertently, by our mis-formed search strategy. Our intended search would only have returned 42% of the relevant articles identified in the search as implemented, for a maximum sensitivity of 0.42 if subsequent citation screening performed perfectly. While the work of human full text screening, these 910 citations would be less than that required for the 5172 citations included by our broader search, combining that broader search with RegEx applied to full text would achieve sensitivity of 0.972 while requiring human full text review of 406 of 5172 citations.

### Automation in *in vitro* systematic reviews

In the first stage, we applied automated full text screening to the results of a search strategy which largely interrogates title and abstract. It is, therefore, likely that additional relevant publications will have been omitted from those search returns, for the same reason as they were not detected by our [TiAb] RegEx. This is confirmed by the very poor performance of what we had considered to be a well-constructed search strategy.

While conceptually attractive, applying the full-text RegEx approach to all of NCBI PubMed is currently impractical, requiring access to the full text of over 30 million scientific publications. We, therefore, explored an intermediate approach, where we trained a machine learning algorithm to detect reports of *in vitro* research, that these might then be interrogated by the full-text RegEx. In a random sample of PubMed Central records, 14.8% included reports of *in vitro* research (based on human full text screening), and the *in vitro* algorithm, applied to Title and Abstract only, performs with sensitivity of 0.954. However, across a corpus of 30m publications, the specificity of 0.85 suggests that of 8.1m publications labelled as reporting *in vitro* research, 3.8m would have been wrongly included, and 200,000 would have been excluded in error.

The performance of the full text RegEx in unselected reports of *in vitro* research is not known, but we estimate a prevalence for inclusion of approximately 0.01% (∼400 from ∼ 4 million). Estimating sensitivity and specificity in this context would require full text screening of several hundreds of thousands of articles and is not currently practicable. However, performance of this approach against the ‘gold standard’ performance identified here, may be feasible. We think that some combination of broad but ‘conventional’ search strategies, combined with algorithmic identification of the *in vitro* literature and RegEx interrogation of selected full-text articles, will prove an effective approach.

## Limitations

Due to lack of full text availability, it was not possible for us to generate a gold standard dataset of all the studies which should be included in the complete corpus of 6232 studies (5172 included in the main analysis + 1060 additional studies). Examining [TiAb] of these additional studies identified an additional 66 potentially relevant studies, but we were not able to confirm this because we were unable to access the full texts. Given a sensitivity for the [TiAb] RegEx of 0.890 as an estimate suggests an additional 10 studies not included by the TiAb RegEx. Taken together, we estimate the total number of relevant studies in the corpus of 6232 to be 76 more than we have identified, suggesting that there are around 395 relevant studies in that corpus.

We can, therefore, provide a rough estimats of the overall sensitivity of various approaches; [TiAb] approaches can be applied to all 6232 and we predict would have identified 332 of the estimated total of 395 studies (sensitivity: 0.841). RegEx [TiAb] would identify 350 (sensitivity: 0.886). Because full-text approaches can only be used where we have access to full text, the sensitivity falls from 316 of 319 to 316 of 395 (human, sensitivity: 0.800) and from 310 of 319 to 310 of 395 (RegEx, sensitivity: 0.785), respectively. Our preferred approach is, therefore, to use full-text RegEx where full text is available, supplemented by [TiAb] RegEx when only abstracts are available. In the example provided, this approach identifies 376 studies (310 from RegEx of full text and 66 from RegEx of [TiAb] when only [TiAb] available). With an estimated 395 relevant studies this represents a sensitivity of 0.952.

One limitation of the RegEx-based approach is that – unlike human screening – it cannot be used where files are not machine readable or where no abstract is provided.

A limitation of the machine learning algorithm for detecting *in vitro* research is that it was trained on only English-language [TiAb]s, and so performance in texts in other languages is not known. Excluding non-English language texts may introduce bias and reduce the generalisability of systematic reviews; although in clinical systematic reviews this has been found to have little or no impact on the conclusions of the review [35], we do not yet know the extent or the impact of this potential bias in reviews of *in vitro* experimental data. The algorithm may also perform poorly in contexts where cell preparations are used as therapies in human studies, for instance CAR-T cells in cancer or stem cell transplantation in neurodegenerative diseases.

## Conclusion

Firstly, we show that in an *in vitro* systematic review, human screening is based on title and abstract erroneously excluded 14% of relevant studies. This may be due to an incomplete description in the abstract of all experiments described in a publication, and this may be more likely in the pre-clinical literature, where several experiments are often presented in a single publication. We then describe a machine learning algorithm which detects publications reporting *in vitro* research with high sensitivity. We propose this tool may be used as a first selection phase in *in vitro* systematic reviews to limit the extent of full-text screening which our first finding suggests is necessary.

## Clinical perspectives

- Systematic reviews of *in vivo* animal experimental data have made important contributions to the evidence-based translation of findings from the laboratory to human clinical trials, and has informed clinical trial design.
- Equally, *in vitro* research makes key contributions to the development of new treatments and therapies.
- Recently, we have seen an increase in the number of systematic reviews investigating *in vitro* research relevant to human health.
- However, the nature of the *in vitro* literature may be different to *in vivo*, and it is important to determine where systematic review methodologies as currently used can be simple applied or may require adaptation.
- Here, we show that title and abstract screening has low sensitivity to identify relevant *in vitro* publications, and we make recommendations to optimise search and screening strategies for *in vitro* systematic reviews.

## Data Availability

Data and code used in the analysis are available on GitHub (https://github.com/emma-wilson/in-vitro-screening) and are shared under a Creative Commons Attribution 4.0 International License. We do not have permission to share the API key required to run the machine learning; however, further information about access is available at: Thomas J, Brunton J, Graziosi S (2010) EPPI-Reviewer 4.0: software for research synthesis. EPPI-Centre Software. London: Social Science Research Unit, Institute of Education.

## Open Access

Open access for this article was enabled by the participation of The University of Edinburgh in an all-inclusive *Read & Publish* agreement with Portland Press and the Biochemical Society under a transformative agreement with JISC.

## Appendix 1 Deviations from protocol

### Method 1: Comparison of screening methods in an example systematic review

- **Full-text PDF retrieval** – Due to time constraints, we did not conduct hand searching for PDFs not retrieved by EndNote X8.
- **Inclusion and exclusion criteria for screening** – Due to the RegEx being written in English, we could only include records with an English-language full text in our analysis. However, our search did not retrieve any records which had to be excluded solely due to this reason.
- **Regular expressions** – Our protocol included both a RegEx for OGD and PC-12 cells. However, we only used the OGD RegEx in our analysis.

### Method 2: Developing a trained machine learning classifier for *in vitro* systematic review screening

- **Risk of bias assessment** – We initially planned to additionally develop a tool to identify risk of bias reporting (randomisation, blinding and sample size calculation) but did not due to time constraints and a lack of studies reporting randomisation, blinding and sample size calculation. Since publishing our protocol, such a tool has been developed for *in vivo* research (Wang et al., 2021b). However it has not yet been validated on *in vitro* research.
- **Supplemented data from NPQIP** – We originally stated we would supplement our machine learning training set with 640 records from NPQIP. This number was written in error, as only 453 records fit our definition of *in vitro* research.

## Appendix 2 Systematic search terms

- **The incorrect strategy implemented in error: fragments containing errors are underlined**
  **NCBI PubMed**
  (‘oxygen–glucose deprivation/reoxygenation’ OR ‘oxygen–glucose deprivation’ OR ‘OGD’ OR ‘OGD/R’ OR ‘oxygen and glucose-deprived model’ OR ‘glutamate’ OR ‘N-methyl-D-aspartate’ OR ‘NMDA’ OR ‘H_2_O_2_’ OR ‘hydrogen peroxide’ OR ‘sodium nitroprusside’ OR ‘SNP’ OR ‘brain ischaemia’ OR ‘brain ischaemia’ OR ‘brain ischemic’ OR ‘brain infarctions’ OR ‘brain infarction’ OR ‘cerebral infarction’ OR ‘cerebral infarction’ OR stroke OR ‘ischemic stroke’ OR ‘neuroprotection’) **AND** ‘PC12’ **OR** ‘PC-12’ **OR** ‘PC 12’
  **Ovid Embase**
  Oxygen–glucose deprivation reoxygenation or oxygen–glucose deprivation or OGD or OGDR or oxygen and glucose-deprived model or glutamate or N-methyl-D-aspartate or NMDA or H_2_O_2_ or hydrogen peroxide or sodium nitroprusside or SNP or brain ischemia or brain ischaemia or brain ischemic or brain infarctions or brain infarction or cerebral infarction or cerebral infarctions or stroke or ischemic stroke or neuroprotection **AND** PC12 **or** PC-12 **or** PC 12
- **The ‘correct’ strategy, only deployed in our analysis of the intended search strategy.**
  **NCBI PubMed**
  (‘oxygen–glucose deprivation/reoxygenation’ OR ‘oxygen–glucose deprivation’ OR ‘OGD’ OR ‘OGD/R’ OR ‘oxygen and glucose-deprived model’ OR ‘glutamate’ OR ‘N-methyl-D-aspartate’ OR ‘NMDA’ OR ‘H_2_O_2_’ OR ‘hydrogen peroxide’ OR ‘sodium nitroprusside’ OR ‘SNP’)
  **AND**
  (‘brain ischaemia’ OR ‘brain ischaemia’ OR ‘brain ischaemia’ OR ‘brain infarction’ OR ‘brain infarction’ OR ‘cerebral infarction’ OR ‘cerebral infarctions’ OR stroke OR ‘ischaemic stroke’ OR ‘neuroprotection’)
  **AND**
  (‘PC12’ OR ‘PC-12’ OR ‘PC 12’)
  **Ovid Embase**
  (Oxygen–glucose deprivation reoxygenation or oxygen-glucose deprivation or OGD or OGDR or oxygen and glucose-deprived model or glutamate or N-methyl-D-aspartate or NMDA or H_2_O_2_ or hydrogen peroxide or sodium nitroprusside or SNP) **and** (brain ischaemia or brain ischaemia or brain ischemic or brain infarctions or brain infarction or cerebral infarction or cerebral infarctions or stroke or ischaemic stroke or neuroprotection) **and** (PC12 or PC-12 or PC 12)

## Appendix 3 Regular expression for oxygen–glucose deprivation

\bOGD\b|(?i)(oxygen|glucose)(\s|-| and)(glucose|oxygen) depriv(ation|ed)|deprived of (oxygen and glucose|glucose and oxygen)

## Abbreviations

API: Application Programming Interface
ASySD: Automated Systematic Search Deduplicator
AUC: Area Under the Curve
CAMARADES: Collaborative Approach to Meta-Analysis and Review of Animal Data from Experimental Studies
EPPI-Centre: Evidence for Policy and Practice Information and Co-ordinating Centre
FPR: False Positive Rate
LDH: Lactate Dehydrogenase
MTT: 3-(4,5-dimethylthiazol-2-yl)-2,5-diphenyltetrazolium bromide
NCBI: National Center for Biotechnology Information
NPQIP: Nature Publication Quality Improvement Project
OGD: Oxygen–Glucose Deprivation
PC-12: Pheochromocytoma-12
PICO: Population, Intervention, Comparator/Control, Outcome
PMC: PubMed Central
PRISMA: Preferred Reporting Items for Systematic reviews and Meta-Analyses
RCT: Randomised Controlled Trial
RegEx: Regular Expression
ROC: Receiver Operating Characteristic
SGD: Stochastic Gradient Descent
SVM: Support Vector Machine
SYRCLE: SYstematic Review Center for Laboratory animal Experimentation
TiAb: Title and Abstract
